# Beluga Optimization Algorithm for Near-Infrared Spectral Variable Selection of Complex Samples

**DOI:** 10.3390/foods14244266

**Published:** 2025-12-11

**Authors:** Javaria Kousar, Liping Yang, Jiale Xiang, Qingwei Mao, Xihui Bian

**Affiliations:** 1 State Key Laboratory of Advanced Separation Membrane Materials, School of Chemical Engineering and Technology, Tiangong University, Tianjin 300387, China; javariakousar66@gmail.com (J.K.); 15222366329@163.com (L.Y.); 17627782013@163.com (Q.M.); 2 School of Pharmaceutical Sciences, Tiangong University, Tianjin 300387, China; 17809277025@163.com; 3 NMPA Key Laboratory for Technology Research and Evaluation of Drug Products, Shandong University, Jinan 250012, China; 4 Key Laboratory of Process Analysis and Control of Sichuan Universities, Yibin University, Yibin 644000, China

**Keywords:** variable selection, beluga whale optimization, partial least squares, spectral analysis, discretization

## Abstract

Near-infrared (NIR) spectroscopy combined with multivariate calibration methods is widely used for the quantitative analysis of complex samples. However, the high-dimensional redundancy of spectra may compromise model predictive accuracy, making it necessary to select variables before modeling. The beluga whale optimization (BWO) algorithm is known for its fast convergence speed, high accuracy and few parameters. The present study employed the discretized BWO (DBWO) algorithm in conjunction with partial least squares (PLS) for spectral quantitative analysis of complex samples. After the optimal number of iterations and transfer function were determined, the PLS models were established based on the randomization test (RT), uninformative variable elimination (UVE) and Monte Carlo uninformative variable elimination (MC-UVE). The predictive performance of DBWO-PLS was compared with full-spectrum PLS, RT-PLS, UVE-PLS and MC-UVE-PLS using wheat, tablet and cocoa bean samples. The results show that all four variable selection methods enhanced model prediction accuracy, with the DBWO-PLS model notably achieving superior performance.

## 1. Introduction

The quantitative analysis of complex samples is essential in various fields, including environmental chemistry, medicine, and food [1]. Nevertheless, in practical applications, complex samples often encompass mixtures of different substances, uncontrolled lighting and intricate features in the spectroscopic signals [2]. These factors make the quantitative analysis of complex samples challenging. Spectral analytical methods are powerful analytical tools that are used in many fields, including food safety, environmental monitoring and medical diagnosis [3]. Among them, near-infrared (NIR) spectroscopy has been increasingly used to analyze complex samples due to its rapid, cost-effective and non-destructive characteristics [4,5,6,7,8]. By constructing quantitative analysis models, the contents of various components in the sample can be determined. However, NIR often exhibits weak signals and significant overlaps, which can increase the complexity of the model [9]. The multivariate calibration technique of chemometrics can determine the content of multiple components without chemical separation [10], effectively avoiding the interference of coexisting components and complex backgrounds in samples [11]. Therefore, multivariate calibration methods are widely used for the quantitative analysis of complex samples.

Many multivariate calibration methods including artificial neural network (ANN) [12], principal component regression (PCR) [13], partial least squares (PLS) [14], support vector regression (SVR) [15], Gaussian process regression (GPR) [16] and extreme learning machine (ELM) [17] have been proposed. Among these methods, PLS is a classical statistical regression model that serves as an effective tool for quantitative prediction [18,19,20,21]. It combines simplicity and good performance for chemical data [22]. However, NIR usually contains thousands of variables, some of which may be irrelevant to the target values. This irrelevance can render the prediction performance of the model unreliable [23,24]. Variable selection methods can extract feature information variables from numerous and complex measurement data, thereby simplifying the multivariate model and even improving its prediction performance [25,26,27]. Therefore, selecting relevant variables is essential before constructing a PLS model.

More and more variable selection methods are being proposed, such as randomization test (RT) [28], uninformative variable elimination (UVE) [29], Monte-Carlo uninformative variable elimination (MC-UVE) [30] and swarm intelligence (SI) optimization algorithms [31]. SI is applied to optimization problems due to its robustness, scalability, generality and flexibility. Beluga whale optimization (BWO) is an SI method known for its strong global search capability and rapid convergence [32,33,34]. This study applies discretized BWO (DBWO) to the spectral variable selection for the first time based on the advantages of the BWO.

In this study, the feasibility of BWO to select spectral variables for the PLS model was discussed using the three near-infrared datasets of wheat, tablets and cocoa beans. Firstly, three transfer functions of V1, V2 and sigmoid were used to discretize BWO, and the optimal transfer function V2 was obtained. Then, the optimal number of iterations of BWO was determined. Finally, the PLS combined with variable selection methods, such as UVE-PLS, MC-UVE-PLS, RT-PLS and DBWO-PLS, were used to establish the model. In the process, the performance of the different regression models was compared. The results demonstrate the robust and competitive performance of the DBWO-PLS model, which achieved high stability with among the smallest number of variables.

## 2. Theory and Algorithm

### 2.1. Beluga Whale Optimization

**Figure 1:**
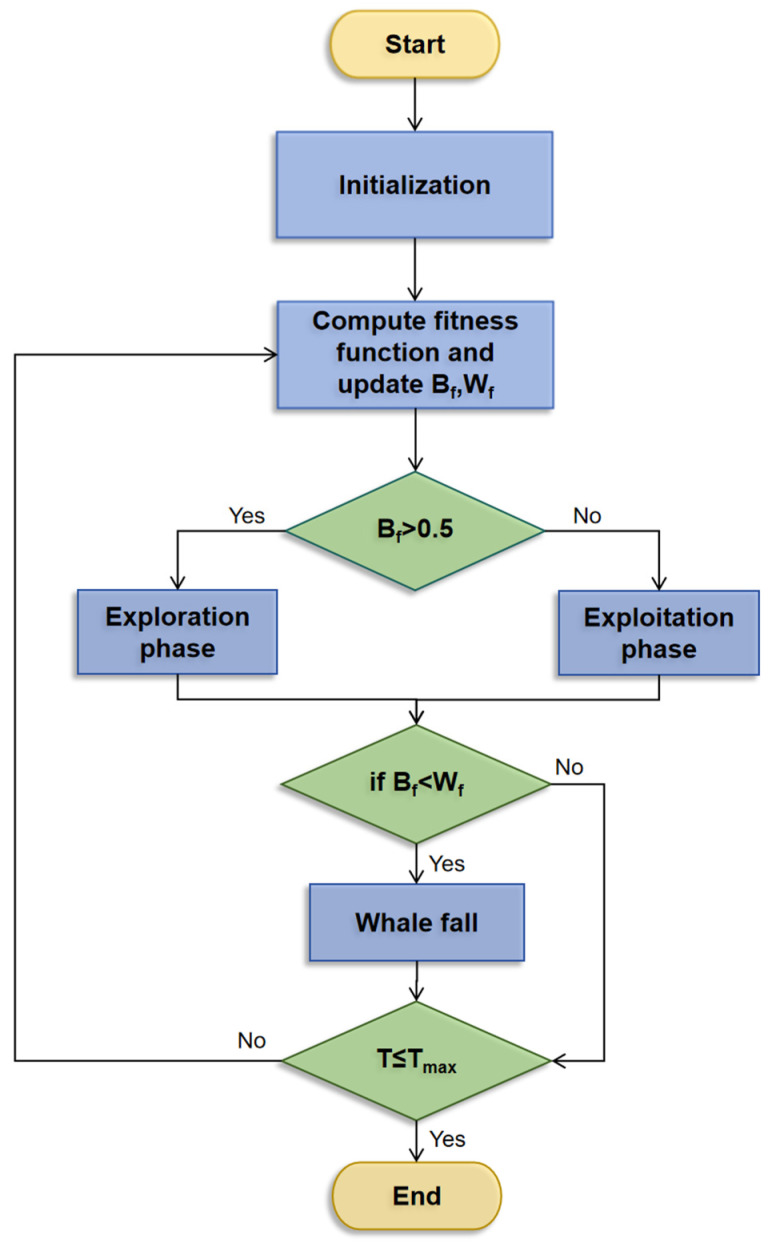
Flowchart of the proposed BWO.

The design inspiration for BWO mainly comes from three typical behavioral patterns of beluga whales, namely swimming, preying and whale fall [32]. Its mathematical model integrates the exploration, exploitation and whale fall phases. The exploration process is designed to simulate the swimming behavior of beluga whales. In the exploration phase, the design space can be explored globally. The exploitation process is designed to simulate the preying behavior of beluga whales. During the exploitation phase, local searches are performed through a random selection of beluga whales. Additionally, during the exploitation process of the algorithm, the Levy flight function was introduced to improve the convergence capability of the algorithm [35]. The schematic diagram of BWO is shown in Figure 1. The balance factor (B_f_) controls the transition between the exploration and exploitation stages in the BWO algorithm. When B_f_ > 0.5, the algorithm transitions into the exploration phase. Otherwise, it proceeds to the exploitation phase. The whale fall probability is denoted as W_f_ and when W_f_ ≥ B_f_, the algorithm proceeds to the whale fall stage.

### 2.2. Chemometric Methods

PLS is a multivariate regression method widely used in spectroscopy, with its core objective being to establish a statistical relationship between two data matrices. This method characterizes the linear relationship between the spectral matrix X and chemical components mathematically by performing orthogonal decomposition on the spectral matrix X and the concentration matrix Y. Latent variables (LVs) are important parameters in PLS regression which significantly influence model performance [36]. An insufficient number of LVs may fail to capture all useful information from the original matrix, while an excessive number can introduce irrelevant noise. Hence, determining the optimal count of LVs is a critical prerequisite for modeling. This study uses the Monte Carlo cross validation (MCCV) method to determine the optimal number of LVs. After calculation, the optimal number of LVs for the wheat, tablet and cocoa bean datasets are 15, 5 and 7, respectively. In addition, this study also uses the UVE, MC-UVE and RT variable selection methods for comparative analysis. The principle of the UVE method is predicated on assessing stability in coefficients estimated by PLS regression. This method introduces a set of random noise variables and sets thresholds based on the distribution of their coefficients, thereby achieving the selection of feature variables. MC-UVE introduces Monte Carlo sampling based on UVE to improve the reliability of stability estimation. It has stronger stability and robustness and is suitable for handling small sample data. RT establishes a null distribution by randomizing the response variable to test the statistical significance of each variable. It is based on the principle of substitution and has both precision and flexibility.

### 2.3. Discretized BWO-PLS

**Figure 2:**
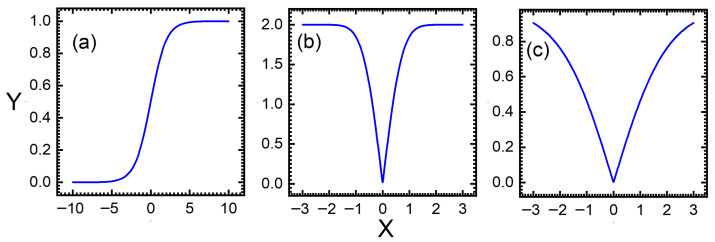
Transfer function curve (the blue curves) of (**a**) sigmoid, (**b**) V1 and (**c**) V2.

The traditional BWO algorithm is mainly used to deal with the problem of continuity optimization. Before applying the BWO to variable selection in NIR spectroscopy, it needs to be converted from a continuous optimization algorithm to a binary one. Therefore, discretization is a key step in variable selection. In this study, the BWO algorithm is discretized by converting the original vectors into binary vectors using different transfer functions. Three transfer functions, namely V1, V2 and Sigmoid, are studied for optimal results. The sigmoid function is a type of S-shaped curve function and its output values are constrained within the range [0, 1]. When the absolute value of the input is very large, the function curve becomes very flat, which makes model training difficult. The V1 function and V2 function are based on the error function and the hyperbolic tangent function, respectively. The calculations of V1 and V2 are very efficient and can effectively train deep networks. The curve variations of the three transfer functions are shown in Figure 2.

As shown in Figure 2, the sigmoid function exhibits an S-shaped monotonic increase. The V1 function and the V2 function are even functions symmetrical about the origin. V2 has a shape similar to V1 but different saturation characteristics. Additionally, the detailed procedures of DBWO-PLS for variable selection can be described as follows.

1. The algorithm initializes a population of beluga whales.
2. For the position of each beluga whale, use the corresponding spectral variables and target vector to construct a PLS model and calculate the fitness.
3. Beluga whale positions are updated in accordance with BWO. Subsequently, a transfer function is applied to convert these updated continuous positions into new binary vectors.
4. The fitness of each beluga whale is re-evaluated based on its new binary position vector after each update. This process continues until the fitness values converge.
5. The binary position vector of the whale with the highest fitness is output as the optimal variable combination. A final PLS model is constructed from this optimal variable subset for predicting the target property.

## 3. Experiments

**Figure 3:**
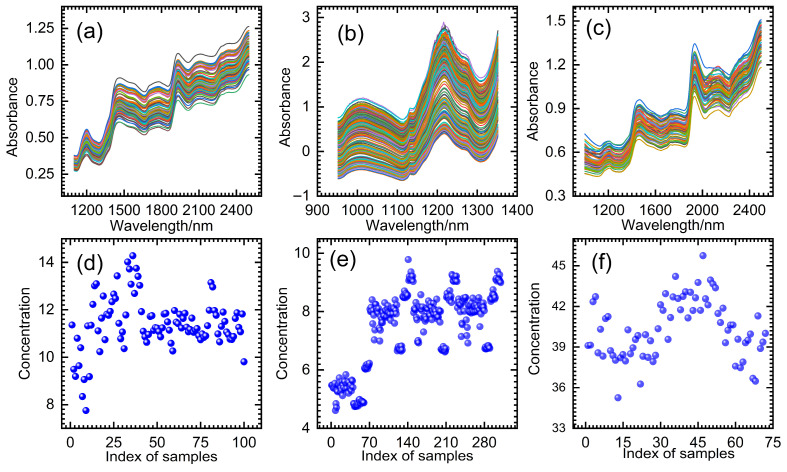
NIR spectra of (**a**) wheat, (**b**) tablets, (**c**) cocoa beans and the content (blue ball) of (**d**) protein, (**e**) active pharmaceutical ingredients and (**f**) fat components, respectively.

The BWO-PLS model was evaluated for its performance using three near-infrared spectral datasets of wheat, tablets and cocoa beans. The wheat dataset was provided by John H. Kalivas [37]. It can be accessed via the FTP server ftp://ftp.clarkson.edu/pub/hopkepk/Chemdata/Kalivas/ (accessed on 6 October 2011). It contains 100 wheat samples with specified protein content. In this study, the NIR spectra of these samples were acquired using diffuse reflectance over a spectral range of 1100–2500 nm at 2 nm intervals. Figure 3a and Figure 3d show the NIR spectra and protein contents measured from the wheat samples, respectively.

The tablet dataset was provided by M. Dyrby et al. [38]. It is available for download via http://www.models.life.ku.dk/Tablets (accessed on 10 April 2024) It contains NIR spectra of 310 samples and the content of active pharmaceutical ingredients. The spectrometer used is the ABB Bomem FT-NIR model MB-160 and the spectral resolution is 16 cm^−1^. The spectra cover a range of 7400–10,507 cm^−1^, which corresponds to 404 predictors. This specific spectral range was used in the experimental analysis. The NIR spectra of the 310 tablet samples and their active pharmaceutical ingredient contents are shown in Figure 3b and Figure 3e, respectively.

The cocoa bean dataset includes NIR spectra of 72 cocoa bean samples and the content of water and fat components. It was provided by Agussabti et al. [39] and can be downloaded at https://data.mendeley.com/datasets/7734j4fd98/1 (accessed on 10 April 2024) In this study, the NIR spectra of the cocoa bean dataset were collected by portable near-infrared spectroscopy (FTIR PSD i15). Spectral data were recorded over the 1000–2500 nm wavelength range, with 32 co-added scans and a spectral resolution of 0.2 nm. The NIR spectra and fat content of the cocoa bean samples are shown in Figure 3c and Figure 3f, respectively.

The three NIR spectral datasets were partitioned into training and prediction sets. The training set was utilized for model construction, while the prediction set was used for external assessment. The study used the Kennard–Stone (KS) grouping method to split the spectral data, with two-thirds comprising the training set and one-third forming the prediction set. For the wheat dataset, there were 67 training samples and 33 prediction samples, respectively. The tablet dataset was partitioned into a training set comprising 207 samples and a prediction set of 103 samples. Based on the KS grouping method, 48 training samples and 24 prediction samples were created from the cocoa bean dataset. However, as shown in Figure 3, the original spectrum contains highly overlapping bands and obvious scattering. Therefore, preprocessing is required before building the model. Various preprocessing methods were studied on the original NIR spectra, such as standard normal variate (SNV), multiplicative scatter correction (MSC) and 1st and 2nd derivatives. Finally, the wheat dataset uses the SNV method, while the tablet and cocoa bean datasets use the MSC method. When comparing the DBWO-PLS, UVE-PLS, MC-UVE-PLS, RT-PLS and single PLS models, the same training set, prediction set and preprocessing method were used.

The performance of the model is assessed using the determination coefficients (R^2^), root mean square error of prediction (RMSEP) and root mean square error of cross validation (RMSECV) in this study. A key criterion for evaluating model performance is a higher R^2^ (up to 1) coupled with lower RMSECV or RMSEP values.

## 4. Results and Discussion

### 4.1. Iteration Number of BWO

**Figure 4:**
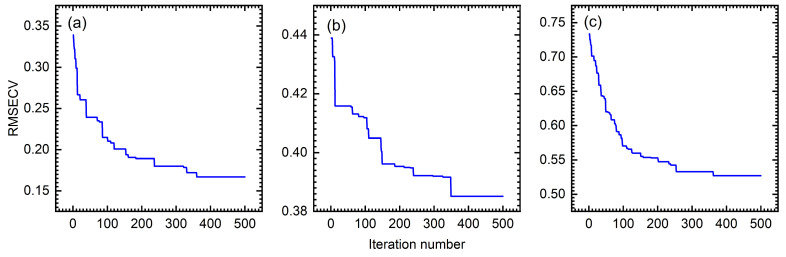
The variation in RMSECV with iteration number in BWO for (**a**) wheat, (**b**) tablets and (**c**) cocoa beans, respectively.

The prerequisite for ensuring algorithm efficiency is determining the appropriate number of iterations. In this study, three datasets were used to execute the DBWO-PLS model. The RMSECV variation with the iteration number for the wheat, tablet and cocoa bean datasets is displayed in Figure 4.

Taking the wheat dataset as an example, the changes in the relevant parameters during the iteration process are presented in Figure 4a. The optimization process can be divided into distinct phases. When the number of iterations is between 0 and 100, the RMSECV value decreases rapidly, indicating that the algorithm is in the optimization process. Subsequently, when the number of iterations is between 100 and 350, the decrease in the RMSECV is significantly slowed. This stage indicates that the performance of the model is gradually converging. Finally, the RMSECV value tends to stabilize when the number of iterations is between 350 and 500. This stage shows that the algorithm has fully converged and approached the optimal solution. A similar trend is observed for the other two datasets. Based on these convergence patterns, the optimal number of iterations is determined to be 500.

### 4.2. Discretization Function

The method of transfer function is applied to realize the discretization of variables in this study. The RMSECV is used as an indicator for evaluating model performance.

**Figure 5:**
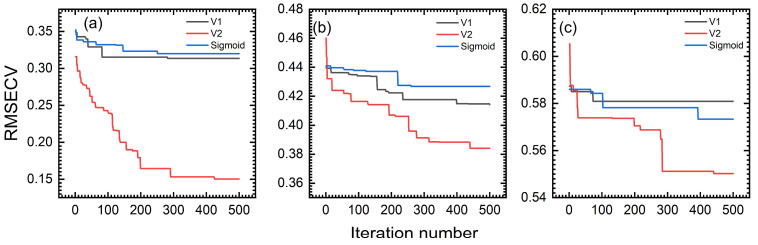
Variation of RMSECV with iteration number by different transfer functions in BWO for (**a**) wheat, (**b**) tablets and (**c**) cocoa beans, respectively.

Figure 5 illustrates the trends of the three transfer functions, which demonstrate similar convergence patterns. As exemplified by the wheat dataset in Figure 5a, the initial RMSECV is relatively high. It quickly decreases as the number of iterations increases, eventually reaching a stable value. Among them, the V2 function exhibits the best performance. Throughout the entire iteration process, it converges the fastest and reaches the lowest RMSECV after stabilizing. Consequently, this study uses the V2 function as the transfer function for the three datasets, which provides a more efficient discretization strategy for the BWO algorithm.

### 4.3. Prediction Results

**Figure 6:**
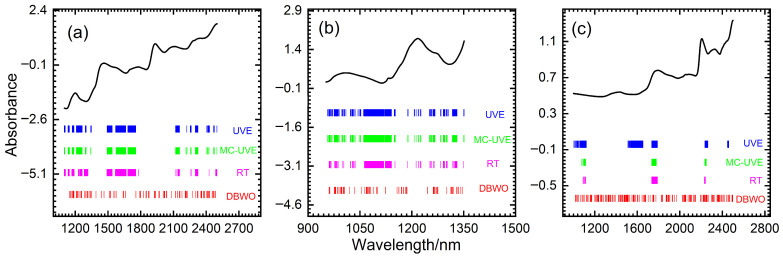
Distribution of variables selected by the four variable methods for (**a**) wheat, (**b**) tablets and (**c**) cocoa beans, respectively.

Based on the optimal parameters, the DBWO algorithm was applied to select variables and establish the PLS model. This study compared the established DBWO-PLS model with RT-PLS, UVE-PLS, MC-UVE-PLS and full-spectrum PLS models to evaluate the efficacy of the DBWO algorithm for variable selection. Figure 6 shows the distribution of selected variables by the four selection methods for the three datasets.

**Figure 7:**
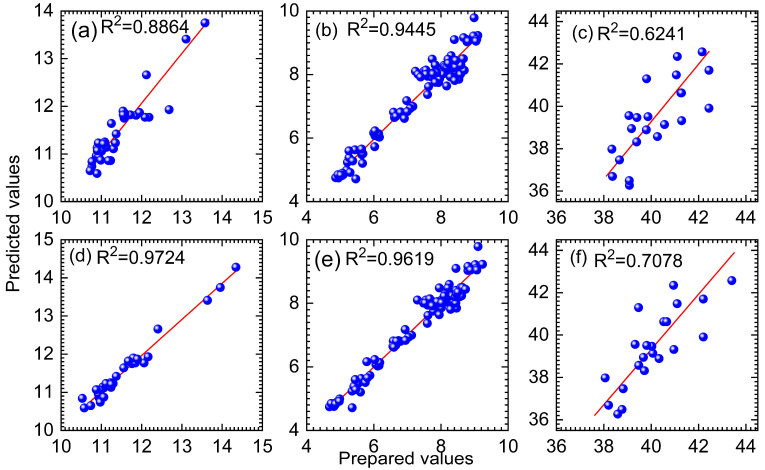
Relationship between the prepared and predicted values by PLS for (**a**) wheat, (**b**) tablets, and (**c**) cocoa beans and by DBWO-PLS for (**d**) wheat, (**e**) tablets, and (**f**) cocoa beans, respectively. (The red line indicates the fitted line).

Analysis of the variable selection results for the wheat and tablet datasets shows that UVE, MC-UVE and RT exhibit obvious localization characteristics. The variables selected by these three methods are mainly concentrated in several specific spectral regions, yielding a significantly large set of variables. In contrast, the variables selected by the DBWO algorithm are fewer and more evenly distributed. This distribution pattern demonstrates that the DBWO algorithm can more effectively identify critical informative variables from different spectral regions. According to the variable distribution results of the cocoa bean dataset, the RT and MC-UVE methods exhibit similar distribution characteristics in variable selection. Although the variables selected by MC-UVE and RT are fewer than those selected by the DBWO algorithm, the chosen variables are mainly concentrated in a few specific spectral regions. Based on the variable distribution results of the three datasets, it can be seen that DBWO is not only capable of efficiently extracting key information for target prediction but also achieving superior prediction performance with the fewest variables. In addition, the performance of the PLS model and the DBWO-PLS model in predicting the target properties of the three datasets is shown in Figure 7.

Figure 7 presents a comparison of the PLS and DBWO-PLS models for predicting target properties for the three datasets. Taking the wheat dataset as an example, the data points in the PLS model are relatively dispersed and clearly deviate from the fitting line. In contrast, the data points in the DBWO-PLS model are distributed more tightly and evenly, showing better predictive performance. In addition, the R^2^ of wheat, tablets and cocoa beans is increased from 0.8864, 0.9445 and 0.6241 to 0.9724, 0.9619 and 0.7078, respectively. This indicates that the predicted values of the DBWO-PLS model have a strong linear correlation with the true values, and its prediction accuracy is much higher than that of the conventional PLS model. The RMSEP values, R^2^ and the number of selected variables for the three datasets are summarized in Table 1 below.

For the wheat dataset, the DBWO-PLS method demonstrates excellent predictive performance. It selected only 87 variables, which was the smallest number selected among all methods. At the same time, it improved R^2^ from 0.8864 to 0.9724 and reduced RMSEP from 0.2846 to 0.1419. Although conventional variable selection methods can achieve variable reduction in feature spaces, their improvement in predictive performance is limited. This highlights the outstanding variable selection capability of the DBWO-PLS method. The DBWO-PLS method also showed the best performance on the tablet and cocoa bean datasets. It selected 55 and 167 variables, respectively, and increased the R^2^ to 0.9619 and 0.7078. Traditional variable selection methods perform similarly to PLS models on tablet data but show limited improvement on the cocoa bean data. This demonstrates that their adaptability to complex matrices is not as good as DBWO-PLS. In conclusion, DBWO-PLS is an effective method for NIR spectral quantitative analysis.

## 5. Conclusions

This study establishes the viability of combining NIR spectroscopy with chemometric techniques for the quantitative analysis of complex samples. A new method combining PLS with DBWO is proposed. After parameter optimization, the PLS, UVE-PLS, MC-UVE-PLS, RT-PLS and DBWO-PLS prediction models were established. The performance of the models was evaluated using three NIR datasets. The results indicate that the DBWO-PLS model significantly improved prediction performance. Compared with the traditional full-spectrum PLS model, its RMSECV decreased by 50.14%, 17.12% and 15.01%, respectively, while the R^2^ were all significantly improved. In comparison with variable selection methods such as UVE-PLS, MC-UVE-PLS and RT-PLS, DBWO-PLS demonstrated overall superiority. It achieves lower RMSECV and higher R^2^ with fewer variables. This indicates that the DBWO algorithm can effectively reduce the complexity of quantitative prediction models. In conclusion, the DBWO-PLS method demonstrates significant potential for the quantitative analysis of complex samples.

## Figures and Tables

**Table 1:** Prediction results of different methods for the three datasets.

Dataset	Methods	Variable Number	RMSEP	R^2^
Wheat	PLS	701	0.2846	0.8864
	UVE-PLS	210	0.3138	0.8619
	MC-UVE-PLS	205	0.3209	0.8556
	RT-PLS	200	0.2975	0.8759
	DBWO-PLS	87	0.1419	0.9724
Tablets	PLS	404	0.3119	0.9445
	UVE-PLS	190	0.3081	0.9458
	MC-UVE-PLS	185	0.3019	0.9455
	RT-PLS	135	0.3023	0.9478
	DBWO-PLS	55	0.2585	0.9619
Cocoa beans	PLS	1557	1.6215	0.6241
	UVE-PLS	290	1.5602	0.6389
	MC-UVE-PLS	75	1.6384	0.6368
	RT-PLS	70	1.5962	0.6357
	DBWO-PLS	167	1.3781	0.7078

## Data Availability

The original contributions presented in the study are included in the article, further inquiries can be directed to the corresponding author.

## Abbreviations

The following abbreviations are used in this manuscript:

NIR	Near-infrared
BWO	Beluga whale optimization
DBWO	Discretized BWO
PLS	Partial least squares
RT	Randomization test
UVE	Uninformative variable elimination
MC-UVE	Monte Carlo uninformative variable elimination
ANN	Artificial neural network
PCR	Principal component regression
SVR	Support vector regression
GPR	Gaussian process regression
ELM	Extreme learning machine
SI	Swarm intelligence
LVs	Latent variables
RMSEP	Root mean square error of prediction
R^2^	Determination coefficients
KS	Kennard–Stone
RMSECV	Root mean square error of cross validation
